# The origin of hydroxy-cyclohexenone fatty acids from skin barrier protein and relevance to covalent binding of ceramides

**DOI:** 10.1016/j.jlr.2025.100843

**Published:** 2025-06-14

**Authors:** Saori Noguchi, William E. Boeglin, Fumie Nakashima, Donald F. Stec, M. Wade Calcutt, Takuya Takeichi, Masashi Akiyama, Alan R. Brash

**Affiliations:** 1 Department of Pharmacology, Vanderbilt University, Nashville, TN, USA; 2 Department of Chemistry, Vanderbilt University, Nashville, TN, USA; 3 Department of Biochemistry, Vanderbilt University, Nashville, TN, USA; 4 Department of Dermatology, Nagoya University Graduate School of Medicine, Nagoya, Japan; 5 Vanderbilt Institute of Chemical Biology, Vanderbilt University, Nashville, TN, USA

**Keywords:** Lipoxygenase, Oxidized lipids, Lipid/chemistry, Sphingolipids, Epoxy fatty acid, SDR9C7, HPLC, mass spectrometry, ^1^H-NMR, UV-Vis spectrometry

## Abstract

Lipid constituents of the skin permeability barrier include a portion of ceramides and fatty acids covalently bound to the barrier protein. The covalent binding requires enzymatic oxidation of linoleate (C18:2) esterified to skin-specific acylceramides, forming a reactive 9,10-epoxy-11*E*-13-keto derivative. Barrier proteins treated with alkali release the bound lipids and as described recently, including two prominent cyclic linoleate derivatives, C18 hydroxy-cyclohexenone fatty acids. Herein we addressed the origin of these cyclic products by alkali treatment of potential precursors. A UV-based assay indicated the rates of Michael adduction of 9,10-epoxy-11*E*-13-keto to cysteine are two orders of magnitude faster than for a typical unsaturated keto fatty acid, and 10-fold faster for the dihydroxy analog, rationalizing their biosynthesis for protein adduction. Alkali treatment degraded the epoxy-ketone and its cysteinyl (glutathione) adduct to multiple UV-absorbing products, although not including the hydroxy-cyclohexenones. By contrast, these derivatives were prominently produced from KOH treatment of the 9,10-dihydroxy-13-ketone or its glutathione adduct. As further evidence of the origin of the hydroxy-cyclohexenones, LC-MS quantitation showed a 90% reduction following KOH treatment of epidermis from mice deficient in Srd9c7, the dehydrogenase in the linoleate oxidation pathway. Taken together, the results confirm the hydroxy-cyclohexenones as derivatives of the linoleate oxidations in the skin barrier pathway and identify the dihydroxy-ketone as a component of the covalently-bonded lipids, and critical to integrity of the epidermal barrier.

The mammalian water permeability barrier in the outer epidermis is a remarkably thin fusion of proteins and lipids that is effective in preserving fluids within the body and obstructing entry of foreign agents from without (1, 2, 3, 4, 5, 6). The anucleated corneocytes in the barrier have the original plasma membrane replaced by a shell of polymerized protein, the corneocyte envelope (CE), and its outside surface is coated with a layer of covalently bound ceramides and hydroxy-fatty acids, the corneocyte lipid envelope (CLE), Fig. 1A (7, 8, 9). Of the many gene products involved in forming the barrier, a subset is critical for preparing and finalizing the covalent binding of lipids to form the CLE. Three enzymes, 12*R*-lipoxygenase (12*R*-LOX), epidermal-lipoxygenase-3 (eLOX3), and short-chain dehydrogenase-reductase family 9C member 7 (SDR9C7), act in series to oxidize linoleic acid (C18:2) esterified in the skin-specific acylceramide Cer-EOS (Esterified Omega-hydroxyacyl-Sphingosine), Fig. 1B (10, 11, 12). Mouse knockout of any one of these three genes almost eliminates covalent binding of the ceramides, with consequent absence of the CLE (10, 12, 13, 14). The mouse gene deficiencies are neonatal lethal due to uncontrolled transepidermal water loss, and in the rare human families with an inactivating mutation, compensation for the water loss results in the hyperproliferative congenital scaly skin disease, ichthyosis (15, 16).

**Fig. 1:**
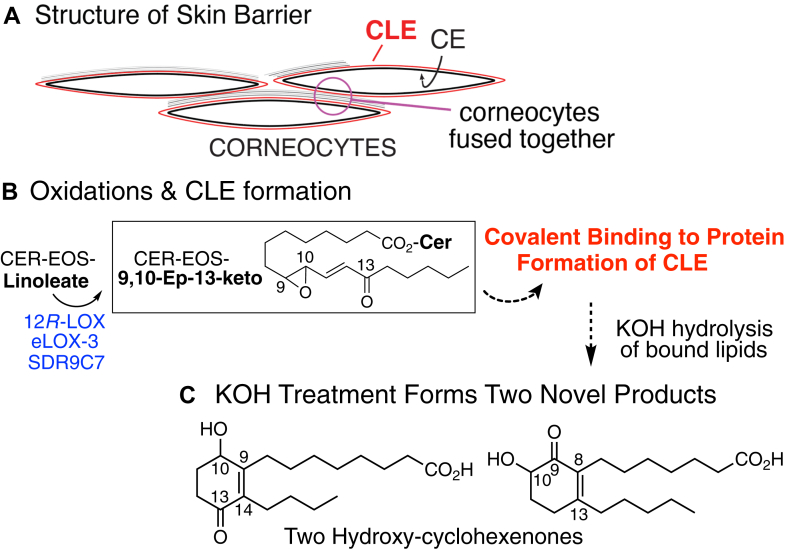
**Structure of the Epidermal Permeability Barrier and Role of 12*R*-LOX Pathway**. A: Corneocytes of the epidermal barrier have a shell of polymerized protein, the CE (corneocyte envelope), comprised of loricrin, involucrin, small proline-rich proteins, periplakin, envoplakin and other minor components (6, 17, 18). The outside surface of the CE has a coating of covalently bound fatty acids and ceramides, the CLE (corneocyte lipid envelope, in *red*), required for barrier function with adjacent corneocytes fused together as CE-CLE/extracellular lipid lamellae/CLE-CE. B: Formation of the covalently bound CLE requires oxidation of linoleate esterified in the acylceramide Cer-EOS by the consecutive actions of the three enzymes 12*R*-LOX, eLOX-3, and SDR9C7 forming the 9,10-epoxy-13-keto derivative, which is capable of non-enzymic covalent attachment to proteins of the CE. C: Mild alkaline hydrolysis of epidermal barrier proteins releases multiple lipids including two hydroxy-cyclohexenones (19), the origin of which is the topic of this manuscript.

The combined activities of the 12*R*-LOX pathway enzymes convert the linoleate esterified in Cer-EOS to a 9*R*,10*R*-*trans*-epoxy-11*E*-13-oxo-octadecenoate, Fig. 1B (10, 11, 12). It is recognized that the chemical structure of this Cer-EOS-epoxy-ketone lends itself to the possibility of covalent coupling to protein, including non-enzymic reactions such as Michael addition and Schiff base adduction to amino acid residues (12). Michael addition is potentially a reversible reaction, and in 2011 an insight into occurrence of this type of linkage was evidenced by the detection of released Cer-EOS-epoxy-ketone from mouse skin barrier proteins (10). Recently, this release of reversibly bound ceramide was used to identify Michael addition to cysteine residues in the barrier protein and to quantify the proportion of covalent binding accounted for by reversible linkages with the conclusion that this mechanism accounts for more than half the total covalent binding in mouse epidermis (20).

A different way to approach the nature of the covalent lipid binding to protein is to clarify the origins of covalently bound lipids that are released from skin barrier proteins by treatment with mild alkali. This methodology has long been employed to quantify the total covalently bound lipids released by KOH-mediated ester bond hydrolysis (21, 22, 23). In recent studies, two unusual components of the lipids released by mild alkali from epidermal corneocytes were identified by UV spectroscopy, mass spectrometry, and ^1^H-NMR as C18 hydroxy-cyclohexenone fatty acids (19), Fig. 1C. These unsaturated C18 fatty acid derivatives are formed in substantial quantity, they are ^14^C-labeled from [^14^C]linoleic acid incubated with mouse skin ex vivo, and the evidence so far suggests they are derived from the covalently bound barrier lipids (19). In this work, we take several approaches to unravel the mechanistic origins of the two C18 hydroxy-cyclohexenone fatty acids and provide evidence that a derivative of the epoxy-ketone end product of the 12*R*-LOX/eLOX3/SDR9C7 pathway is a major species of covalently bound lipid in the epidermal barrier.

## Materials and methods

### Preparation of 9,10-(*trans* or *cis*)-epoxy-11*E*-13-oxo-octadecenoic acid

13*S*-Hydroxy-9*Z*,11*E*-octadecadienoic acid (13*S*-HODE) was prepared in 200 mg batches by transformation of linoleic acid (NuChek Prep Inc., Elysian, MN) with soybean lipoxygenase (Sigma, type V) followed by reduction with sodium borohydride as described (24). 13*S*-HODE (50 mg in 1 ml DCM) was cooled on ice and oxidized by treatment with 1 equivalent of DDQ, (2,3-dichloro-5,6-dicyano-1,4-benzoquinone), the immediate reaction turning the bright orange reagent to brown. The sample was diluted to 2 ml with DCM and applied to a 1 g Bond-Elut silica cartridge (Agilent); after adding two 1 ml DCM rinses of the vial the cartridge was eluted with 8 ml DCM/MeOH/HAc (100:1:0.1 by volume), yielding 32 mg 13-oxo-ODE by UV analysis (λmax 280 nm, ε = 25,000 M^-1^ cm^−1^); 2 ml further elution gave a mixture of 13-oxo-ODE (7 mg) and 13-HODE. To affect isomerization of 13-oxo-ODE to 9*trans*,11*trans*-13-oxo-ODE, with the sample dissolved in 5 ml 20% ethyl acetate in hexane, 2.5 μl of a 1 mg/ml solution of iodine in hexane was added and the sample exposed for 4 min to daylight (25). The iodine was then reduced to iodide by vigorous mixing with 2 mg/ml aqueous sodium sulfite, the aqueous phase was discarded and the organic phase washed twice with water and taken to dryness under a stream of N_2_. Normal-phase HPLC analysis typically showed ∼10:1 ratio of *trans-trans* to *cis-trans* 13-oxo-ODE (solvent hexane/IPA/HAc (100:0.5:0.02 by volume). Selective epoxidation of the 9,10 double bond was effected using meta-chloroperoxybenzoic acid (mCBPA), 1.5 equivalents in DCM; this reaction was relatively sluggish and the sample was left to react overnight at room temperature then diluted with additional DCM and extracted twice with 1M K_2_HPO_4_ to remove most of the remaining mCPBA and mCBA by-product (5 min spin at 2000 RPM was required to separate the phases). After washing the organic phase with water, it was taken to dryness under N_2_.The major 9,10-*trans*-epoxy-11*E*-13-ketone and the corresponding 9,10-*cis* epoxy isomer were separated and purified using a semi-preparative Beckman 5 μ silica column (25 × 1 cm) with a solvent of hexane/IPA/HAc (100:2:0.02 by volume); at a flow rate of 4 ml/min, unreacted 13-oxo-ODE eluting at 7.4 min, the *trans*-epoxy-ketone eluting at 9.0 min and the *cis-*epoxy-ketone isomer at 11.2 min. The epoxy-ketones were quantified by UV analysis using ε = 12,500 M^-1^ cm^−1^ for the conjugated enone (26), λmax 233 nm in ethanol for 9,10-*trans*-epoxy-11*E*-13-ketone, and 235 nm for the *cis*-epoxy isomer, overall yield typically 15% of the major isomer. NMR of 9,10-*trans*-epoxy-11*E*-13-keto-octadecenoate methyl ester, C_6_D_6_, δ 6.45 1H, dd, J_10,11_ = 7.0, J_11,12_ = 15.9 Hz, H11; 6.235, 1H, d, J_11,12_ = 15.8 Hz, H12; 3.355, 3H, s, CH_3_O; 2.80, 1H, dd, J_9,10_ = 1.5 Hz, J_10,11_ = 6.9 Hz, H10; 2.499, 1H, dt, J_9,10_ = 1.9 Hz, J_8,9_ = 5.5 Hz, H9; 2.157, 2H, t, J = 7.3 Hz, H14; 2.109, 2H, t, J = 7.4 Hz, H2; 1.55, 4H, m, H3 and H15; 1.29, 2H, m, H8?; 1.23–1.09, 12H, m, H4, 5, 6, 7, 16, 17; and 0.835, 3H, t, J = 7.2 Hz, H18.

### Preparation of 9,10-dihydroxy-11E-13-oxo-octadecenoic acids

Recombinant soluble epoxide hydrolase (sEH) was prepared as described previously (27). The enzyme was used to hydrolyze the epoxy functionality of 9,10-*trans*-epoxy-ketones to the corresponding 9,10-*erythro*-dihydroxy product with reversal of configuration at the 10-carbon. In a typical preparation, 300 μg of epoxy-ketone (or its methyl ester) is taken to dryness in a 5 ml Reactivial dissolved in 5 μl ethanol, and the following added with mixing: 900 μl 0.1 M Tris buffer pH 8 containing 500 mM NaCl, 50 μl of 10 mg/ml gelatin (the added protein helps stabilize sEH), and 10 μl (56 μg) sEH with incubation for 60 min at room temperature. (A small aliquot is examined on RP-HPLC after 30 min to monitor the progress of the hydrolysis). The solution is then adjusted to pH 4 (not required for reactions with methyl ester substrate) and extracted thoroughly with 3 ml dichloromethane. The vial is centrifuged at 2,000 RPM to separate the phases, the organic solvent transferred to a fresh vial and evaporated to dryness, and the sample dissolved in RP-HPLC solvent. The dihydroxy-ketone product is purified using an Agilent Eclipse 5 μ XDB-C18 column (15 × 0.46 cm) with a solvent of acetonitrile/water/glacial acetic acid in the proportions 70:30:0.01 for methyl esters (at a flow rate of 1 ml/min the dihydroxy-ketone and epoxy-ketone methyl esters elute at 2.9 and 8.6 min, respectively) and adjusted to the proportions 60:40:0.01 for the free acids. [1–^14^C]9,10-*erythro*-Dihydroxy-11*E*-13-oxo-octadecenoic acid was prepared through the above routes starting with 30 μCi [1–^14^C]linoleic acid admixed with 5 mg cold linoleic acid, with an 8% yield of purified radiolabeled dihydroxy-ketone. The conjugated enone chromophore of dihydroxy-11*E*-13-oxo-octadecenoate exhibits a λmax at 226 nm in ethanol and was used to quantify the product using the same molar extinction coefficient measured for the epoxy-ketone, 12,500 M^-1^ cm^−1^ (26). 9*R*,10*S*-dihydroxy-11*E*-13-oxo-octadecenoate methyl ester, C_6_D_6_, δ 6.77, dd, 1H, (*J*_*11,12*_ = 15.9, *J*_*10,11*_ = 5.0 Hz), H11; 6.33, dd, 1H, (*J*_*11,12*_ = 15.9 Hz, *J*_*10,12*_ = 1.7 Hz), H12; 3.83, m, 1H, H10; 3.35, s, 3H, OCH_3_; 3.33, m, 1H, H9; 2.24, t, 2H, (J = 7.3 Hz), H14; 2.10, t, 2H, J = 7.4 Hz), H2; 1.71 (weak), m, H9-OH; 1.60, m, 3H, H15, H10-OH; 1.53, p, 2H, H3; 1.35-1.22, m, 2H, H8; 1.22-1.05, 10H, H17, 4, 5, 6, 16; 0.83, t, 3H, H18.

### Assignment of chirality of 9,10-*trans*-epoxy-11*E*-13-oxo-octadecenoates

By chiral HPLC as the free acid, racemic 9,10-*trans*-epoxy-11*E*-13-ketone is well resolved using a Lux® Amylose-1 column (Phenomenex, 3 μm, 25 × 0.46 cm) with a solvent of hexane/EtOH/MeOH/glacial acetic acid (100:5:5:0.01 by volume) and a flow rate of 1 ml/min, with retention times 43.4 and 50.6 min, although the order of elution of the enantiomers remained to be established. (Separation of the corresponding methyl esters on the chiral column is minimal and not suitable for preparative resolution). To assign chirality, the first-eluting enantiomer from the chiral column was reduced with NaBH_4_ and the two epoxy-alcohol diastereomers separated by normal-phase HPLC. From previous work it is established that the first-eluting diastereomer from the NP-HPLC silica column is *RRR* (9*R*,10*R*,13*R*) or *SSS* in chirality, and the second-eluting isomer is *RRS* or *SSR* (28). Furthermore, chiral HPLC of the *RRR/SSS* enantiomers using a Chiralpak AD-H column (or its equivalent Amylose-1) resolves the methyl esters in the order *RRR* eluting before *SSS* (10). Accordingly, the unknown chiralities of the parent epoxy-ketones could be established by chiral HPLC of the first epoxy-alcohol diastereomer from NP-HPLC. Chiral HPLC analysis in comparison to authentic *RRR* and *SSS* epoxy-alcohol standards showed that the first-eluting diastereomer from NP-HPLC co-chromatographed with the second (*SSS*) enantiomer on the chiral column. This experiment was repeated using the exact same Chiralpak AD-H column used in the original reports (10, 28), and confirming the above orders of elution.

The results establish that while the epoxy-alcohols chromatograph in the order *RRR* before *SSS* by chiral chromatography (10), the parent 9,10-*trans*-epoxy-ketones chromatograph in the order *SS* before *RR* on the same chiral HPLC column. Apparently, upon reduction of the epoxy-ketones to epoxy-alcohols, the *R/S* chirality of the 13-hydroxyl has a dominating influence and reverses the order of elution of the epoxide functionality. Thus, on the Amylose-1 or Chiralpak AD-H chiral columns, the 9*S*,10*S*-11*E*-13-oxo-octadecenoate elutes before the 9*R*,10*R*-11*E*-13-oxo enantiomer. Using this knowledge and a semi-preparative Amylose-1 column (5 μ, 25 × 1 cm), the epoxy-ketone enantiomers were resolved and made available on a milligram scale.

### Ultraviolet Spectroscopy (UV), rates of reaction

Reactions with amino acids were quantified using an Agilent Technologies Cary 60 UV-Vis spectrometer as the rate of decrease in UV absorbance recorded at the λmax values of the conjugated enone chromophores of linoleate epoxy-ketone and dihydroxy-ketone (235 nm and 228 nm, respectively, in 0.1 M potassium phosphate). UV cuvettes contained 0.485 ml potassium phosphate (0.1 M, pH 7.5) and 5 μl linoleate epoxy- or dihydroxy-ketones in methanol (100 μM final concentration, quantified using a molar extinction coefficient of 12,500 M^-1^ cm^−1^); reaction was initiated by addition of 10 μl 50 mM amino acid (1 mM final concentration) with recording of absorbance for up to 10 min. Measurements at pH 8.5 were recorded at the relatively high wavelength of 240 nm to avoid UV saturation effects due to the stronger absorbance of cysteine at pH 8.5. To correct these 240 nm absorbance readings to values at the respective λmax, the measured rates were adjusted using a factor of 1/0.94 for the epoxy-ketone and 1/0.67 for the dihydroxy-ketone as the respective λmax/240 nm ratios. Assay of 13-oxo-ODE with cysteine used similar methodology except monitoring absorbance at the λmax of 280 nm and using a molar extinction coefficient of 25,000 M^-1^ cm^−1^.

### Preparation and purification of thiol adducts

Reactions with 25–100 μg/ml linoleate conjugated enones with cysteine, glutathione, or N-acetyl-cysteine methyl ester were conducted in 0.1 M potassium phosphate (pH 7.5) and monitored by UV spectroscopy (disappearance of the conjugated enone chromophore) by repetitive scanning over 350–200 nm using a Cary 60 UV-Vis spectrometer (Agilent). Reaction of the epoxy-ketone methyl ester (200 μg) with 2 μmole lysine was conducted in 100 μl acetonitrile/pH 10 borate buffer (2:1) as described (29) and analyzed by HPLC-UV, proton NMR and mass spectrometry. An aliquot of epoxy-enone methyl ester and lysine reaction mixture was analyzed on a Waters Symmetry® C18 5 μm column (25 × 0.46 cm) with isocratic elution using MeOH/10 mM Ammonium acetate (80:20 by volume) with a flow rate of 1 ml/min with diode array UV detection at 205, 220, 235 and 270 nm. Analysis of pyrrole adduct of lysine with 9,10-*trans*-epoxy-11*E*-oxo-octadecenoate substrate was performed by LC-MS-ESI (negative ion) analysis, with the fatty acid pyrrole adduct with [M-H]^-^ ion at *m/z* 451. For extraction, the solutions were acidified to pH 5 and applied to a pre-equilibrated Oasis cartridge (Waters), 30 mg size for 1–2 ml volumes or 60 mg Oasis cartridge for up to 10 ml. After loading and washing the cartridge with water, products were eluted with 70:30 acetonitrile/water or methanol.

Conjugates of the epoxy-ketone methyl ester with N-acetyl cysteine methyl ester used conventional solvents: for RP-HPLC, acetonitrile/water/glacial acetic acid (60:40:0.01 by volume), a flow rate of 0.5 min/min with retention times of the two thiol diastereomers at 30.4 and 31.7 min. The two diastereomers were further purified individually using a Thomson 5 μ silica column (25 × 0.46 cm) with a solvent of hexane/isopropanol (100:10 by volume), a flow rate of 0.5 ml/min with a retention time of the conjugates of 22 min (the two diastereomers co-chromatograph on NP-HPLC).

### Quantitative LC-MS analysis of 10-hydroxy-13-oxo-cyclohexenone C18:1 in Sdr9c7 epidermis

(Animal care and experimental procedures were approved by the Animal Experiment Committee, Graduate School of Medicine, Nagoya University, and were conducted according to the Regulations on Animal Experiments of Nagoya University). Mouse pup epidermis, five of each *Sdr9c7* genotype (12) were shipped from Japan in ethanol and stored at −70 °C until analysis. Samples were transferred to a 5 ml vial, ethanol removed, and replaced with 3 ml CHCl_3_/MeOH (2:1), and the samples processed essentially as described before (ref (12), Supplement) with additional extractions of the protein pellet, all performed at 4°C and under argon. Briefly, after the first overnight soak, 1 ml of MeOH was added (making 1:1 CHCl_3_/MeOH), the samples were diced, homogenized (Sonic Dismembrator Model 100 with 2 mm probe), centrifuged (3000 x g, 15 min), the supernatant removed, and the pellet resuspended and washed four more times overnight with CHCl_3_/MeOH as above. The pellets were suspended in 1 ml 1N KOH in 95% methanol and stirred at room temperature overnight under argon. The samples were acidified to pH 4–pH 5 and extracted using the Bligh and Dyer protocol (30). The organic phase was taken to dryness and stored in MeOH at – 70°C. Prior to LC-MS, the samples were transferred to column solvent and analyzed using a Thermo Q Exactive HF hybrid quadrupole/orbitrap high resolution mass spectrometer instrument on a Waters Symmetry 5 μ C18 column (150 × 2.1 mm) with an isocratic solvent of CH_3_CN/H_2_O/glacial acetic acid (50:50:0.01 by volume) at a flow rate of 0.3 ml/min. The instrument was operated in full scan, negative ion APCI, with monitoring of the M-1 ion for 10-hydroxy-13-oxo-cyclohexenone C18:1 (*m/z* 309.2055) and M-18 (291.1954). Peak areas were calibrated versus injections of authentic standard quantified based on UV absorbance (λmax 245 nm in MeOH, ε = 12,500).

### LC-MS methods

LC-MS analyses used a TSQ Vantage triple quadrupole mass spectrometer for product identification and quantitative analyses and a Thermo Q Exactive HF hybrid quadrupole/orbitrap for high resolution MS. The TSQ Vantage triple quadrupole mass spectrometer (Thermo Scientific) is equipped with an Ion Max API source, a HESI-II probe, and a 50 μm ID stainless steel high voltage capillary. Data acquisition and analysis were carried out using Xcalibur v.2.1.0, Vantage v.2.3.0 and LCQuan v.2.7.0 software (Thermo). A Kinetex C18 analytical column (100 × 3 mm), 2.6 mm particle size, (Phenomenex) was used for all chromatographic separations. Sample analyses were carried out using a Waters Acquity UPLC system (Waters), made up of a binary solvent manager, refrigerated sample manager, and a heated column manager.

High-resolution LC-MS analysis used the Thermo Q Exactive HF hybrid quadrupole/orbitrap high resolution mass spectrometer interfaced to a Vanquish Horizon HPLC system (Thermo Fisher). For product identification, the mass spectrometer was operated in alternating positive and negative ion mode, with targeted selected ion monitoring detection of specified precursor ions at a resolving power of 30,000, an isolation window of 2.0 *m/z*, and the following HESI source parameters: spray voltage 4 kV; capillary temperature 300°C; HESI temperature 100°C; *S*-lens RF level 50; N_2_ sheath gas 40; N_2_ auxiliary gas 10; sweep gas 2.0; in-source CID off. Data acquisition and quantitative spectral analyses were done using Thermo Xcalibur version 4.1.31.9 and Thermo LCQuan version 2.7, respectively.

### NMR analyses

^1^H NMR and ^1^H,^1^H COSY NMR experiments were acquired using a 14.0 T Bruker magnet equipped with a Bruker AV-III console operating at 600.13 MHz. Spectra were acquired in 3 mm NMR tubes using a Bruker 5 mm TCI cryogenically cooled NMR probe in benzene-*d*_6_ and chemical shifts referenced to benzene, 7.16 ppm.

## Results

### Conjugation with amino acids, UV-assay, rates of reaction

We noted earlier that formation of the conjugated enone moiety of 9,10-*trans*-epoxy-11*E*-13-oxo-octadecenoate on Cer-EOS molecules presents the possibility of covalent coupling reactions with lysine, histidine or cysteine residues on skin barrier proteins (12). The transformations of linoleate epoxy-ketones are reported in detail for reaction with histidine (31) or lysine (29). We prepared a potential lysine conjugate with 9,10-*trans*-epoxy-11*E*-13-oxo-octadecenoic acid, a pyrrole, and characterized its UV signature as a smooth distinctive chromophore λmax 229 nm, completely different in profile from the linoleate epoxy-ketone, its molecular mass by LC-MS (*m/z* 451, [M-H]^-^), and its proton NMR spectrum recorded in d_4_-methanol with the characteristic pyrrole protons at 5.72 and 5.93 ppm (Supplemental Figs. S1 and S2).

**Fig. 2:**
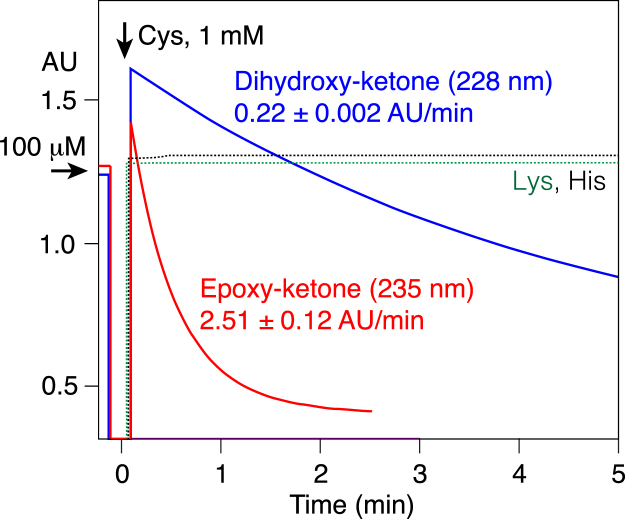
**Rates of reaction of linoleate epoxy-ketone and dihydroxy-ketone with amino acids**. Reactions were conducted in 0.1 M potassium phosphate pH 7.5 at the respective λmax, 235 nm for 9,10-*trans*-epoxy-11*E*-13-oxo-octadecenoic acid (red trace) and 228 nm for 9,10-dihydroxy-11*E*-13-oxo-octadecenoic acid (*blue*). Initial rates after addition of 1 mM cysteine (final concentration) were, respectively, 2.51 ± 0.12 AU/min (SEM, n = 6) and 0.22 ± 0.002 AU/min (n = 3). Lysine (1 mM, *dotted green trace*) and histidine (1 mM, *dotted black*) gave no observable reaction with epoxy-ketone (or dihydroxy-ketone, not illustrated) over the time course.

Nonetheless, as reported (20), in comparison to cysteine, both histidine and lysine react sluggishly with linoleate epoxy-ketone. We utilized a UV absorbance assay (Experimental Procedures) to quantify the rates based on loss of the conjugated enone chromophores of 9,10-*trans*-epoxy-11*E*-13-ketone and 9,10-*erythro*-dihydroxy-11*E*-13 ketone upon adduction with amino acids, Fig. 2. The oxidized linoleates (100 μM) react in seconds with 1 mM cysteine, with rates at pH 7.5 of 2.51 ± 0.12 AU/min with the epoxy-ketone (equivalent to 201 μM/min) and about ten-fold lower, 0.22 ± 0.002 AU/min, with the dihydroxy-ketone (18 μM/min), while there was no observable reaction with histidine or lysine, Fig. 2. These nonenzymic reactions are strongly pH-dependent, related to the abundance of the thiolate anion and its pKa of ∼ 8.6 in aqueous solution (32), and accordingly, the rates are higher at pH 8.5 (Supplemental Fig. S3), and approximately ten-fold lower at pH 6.5 compared to pH 7.5 Both the epoxy-ketone and dihydroxy-ketone react much faster with cysteine than does the prototypical unsaturated keto fatty acid 13-oxo-9*Z*,11*E*-octadecadienoic acid (13-oxo-ODE); in molar terms, its rates at pH 7.5 (measured at its λmax of 280 nm) were 0.3% and 3% of the rates with epoxy-ketone and dihydroxy-ketone, respectively, and earlier reports indicate it requires pH 10 to react 100 μM concentrations of 13-oxo-ODE within an hour at room temperature (33, 34).

### Assignment or product structures by LC-MS and NMR

The thiol adducts were analyzed by LC-MS by high-resolution negative ion ESI. The two diastereomers of the cysteinyl adduct of the 9*R*,10*R*-epoxy-13-ketone gave the [M-H]^-^ ion as the base peak at *m/z* 430.2258 (calc. 430.2269), Supplemental Fig. S4. The cysteinyl adducts of 9,10-dihydroxy-13-ketone also have the [M-H]^-^ ion base peak recorded at *m/z* 448.2375 (calc. 448.2374), not shown. Masses for the glutathione adducts were also confirmed with [M-H]^-^ ions at *m/z* 616.2892 (calc. 616.2904) for the epoxy-ketone adduct and *m/z* 634.2995 (calc. 634.3015) for the dihydroxy-ketone, all these values within 3 ppm of the predicted masses.

**Fig. 3:**
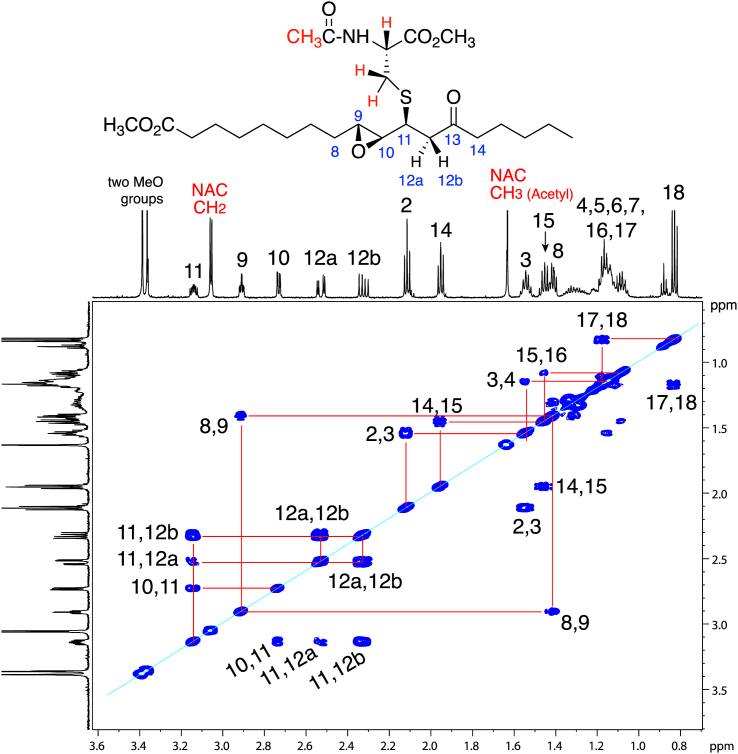
**^1^H-NMR spectrum and COSY analyses of a thiol conjugate of 9*R*,10*R*-*trans*-epoxy-11*E*-13-ketone.Partial 600 MHz spectrum (0.7–3.6 ppm) and COSY of the first-eluting (RP-HPLC) diastereomeric conjugate of 9*R*,10*R*-*trans*-epoxy-11*E*-13-oxo-octadecenoate methyl ester with the methyl ester of N-acetyl-cysteine recorded in d_6_-benzene.** Thiol adduction at C11 of the epoxy-ketone is clearly evidenced. Not included in this partial spectrum are two protons further downfield, the NAC main chain proton, a double triplet at 4.90 ppm, and the NAC NH proton, a doublet at 6.34 ppm; the spectrum of the second-eluting diastereomer is illustrated in Supplemental Fig. S5.

On purely chemical grounds, the thiol adduction to 9,10-epoxy/dihydroxy-11*E*-13-oxo structures should be on the 11-carbon. A clear and unambiguous assignment of adduction at C11 was most apparent on the structure of the diastereomers of the conjugates of the chiral epoxy-ketone methyl ester with N-acetyl cysteine methyl ester, Fig. 3 and Supplemental Fig. S5. Use of the methyl ester derivatives allowed a second round of purification by NP-HPLC and solubility in a non-polar solvent (d_6_-benzene) for recording the NMR spectra. This produce well resolved and distinct signals linking H11 to its neighboring H10 and H12 protons and coupling between the protons on the 8- through 12-carbons, (with the absence, as is typical, of a 9,10 cross-peak across the *trans*-epoxide), Fig. 3 and Fig. S5.

### Effects of mild alkali treatment of the epoxy-ketone and dihydroxy ketone

**Fig. 4:**
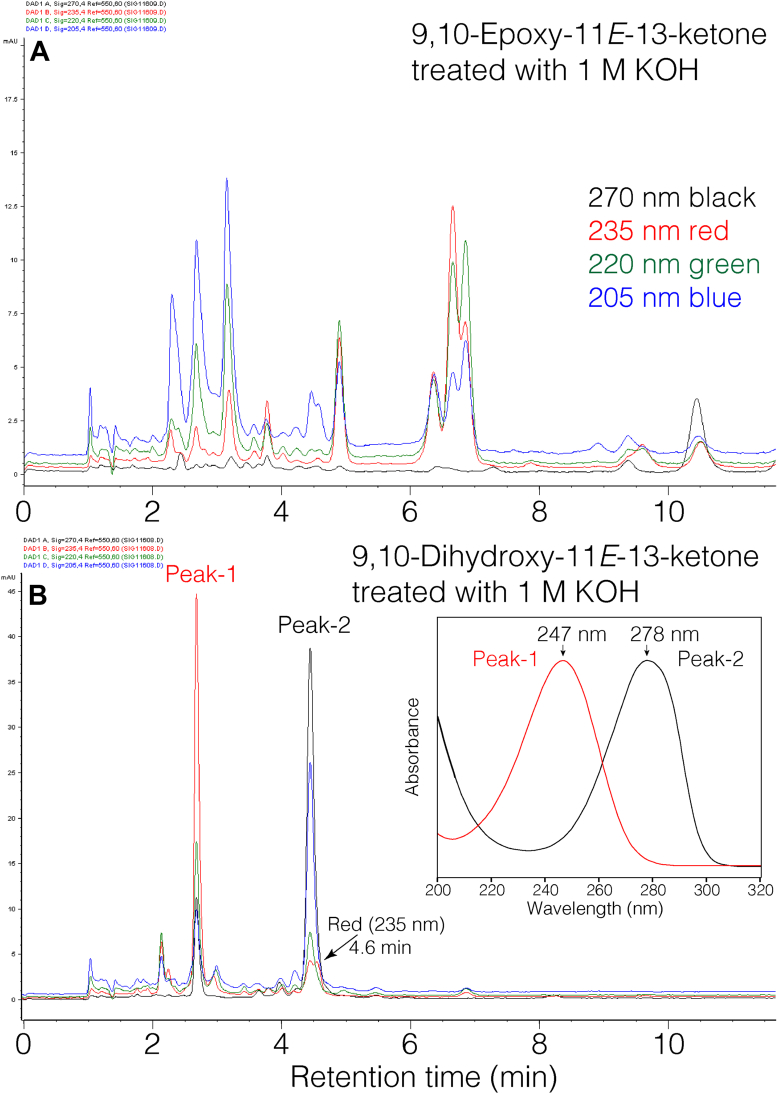
**RP-HPLC analysis of products of 9,10-*trans*-epoxy-11*E*-13-ketone and 9,10-*erythro*-dihydroxy-11*E*-13-ketone after treatment with 1M KOH.** A: Product profile from alkali treatment of 9,10-*trans*-epoxy-11*E*-13-oxo-octadecenoic acid analyzed using an Agilent Eclipse 5 μ XDB-C18 column (15 × 0.46 cm) with a solvent of CH_3_CN/H_2_O/HAc (60:40:0.01 by volume) at a flow rate of 1 ml/min. B: The next injection on the column under the same conditions shows the product profile from alkali treatment of 9,10-*erythro*-dihydroxy-11*E*-13-oxo-octadecenoic acid. Four channels of diode array UV detection represent 270 nm (*black*), 235 nm (*red*), 220 nm (*green*) and 205 nm (*blue*).

In an earlier publication, we showed that two hydroxy-cyclohexenones are produced by alkali treatment of covalently bound lipids of skin barrier proteins, and both metabolites were ^14^C-labeled following incubation of [^14^C]linoleic acid with mouse skin ex vivo (19). Here we further addressed their mechanistic origin by KOH treatment of their potential precursors, the end products of the 12*R*-LOX pathway, linoleate 9,10-*trans*-epoxy-11*E*-13-ketone and its 9,10-dihydroxy hydrolysis product. As indicated by RP-HPLC analysis, treatment of the epoxy-ketone with 1 M KOH in 95% MeOH overnight at room temperature resulted in its complete degradation to a complex mixture, Fig. 4A. The nature of the breakdown products was not pursued here, because the main question was the origin of the hydroxy-cyclohexenones, and there was absence of either from the epoxy-ketone.

The treatment of the dihydroxy-ketone gave two major UV-absorbing products on RP-HPLC designated as Peak-1 and Peak-2, Fig. 4B. The earlier eluting Peak-1 was identified from its HPLC retention time, UV spectrum (Fig. 4B, inset), molecular weight (LC-MS, [M-H]^-^ ion at *m/z* 309), and comparison with the authentic standard as the 10-hydroxy-13-oxo-cyclohexenone, originally termed the “polar” lipid product produced by KOH treatment of skin barrier proteins (19).

Peak-2, the later eluting prominent product on RP-HPLC showed a UV spectrum with λmax 278 nm, and in contrast to the typically smooth and symmetrical appearance of the UV spectrum of conjugated enone Peak-1, the chromophore of Peak-2 has a slight and characteristic asymmetry (the absorbance decreases more abruptly on the high wavelength side), (Fig. 4B inset). Analysis by LC-MS using negative ion electrospray gave an [M-H]^-^ ion at *m/z* 321, indicating a molecular mass of 322, 6 a.m.u. lower than the dihydroxy-ketone starting material and 12 a.m.u. more than hydroxy-cyclohexenone Peak-1. Structural assignment of Peak-2 was aided greatly by finding it is an oxidation product of the second of the original pair of hydroxy-cyclohexenones, the 9-oxo-10-hydroxy isomer, previously termed the “less polar” lipid (19). On RP-HPLC the two almost co-chromatograph, and in Fig. 4B the cyclohexenone is detectible as a minor peak in the 235 nm channel underneath the back shoulder of Peak-2 and labeled with an arrow at a retention time of 4.6 min. KOH treatment of authentic 9-oxo-10-hydroxy-cyclohexenone produced Peak-2 (Supplemental Fig. S6).

**Fig. 5:**
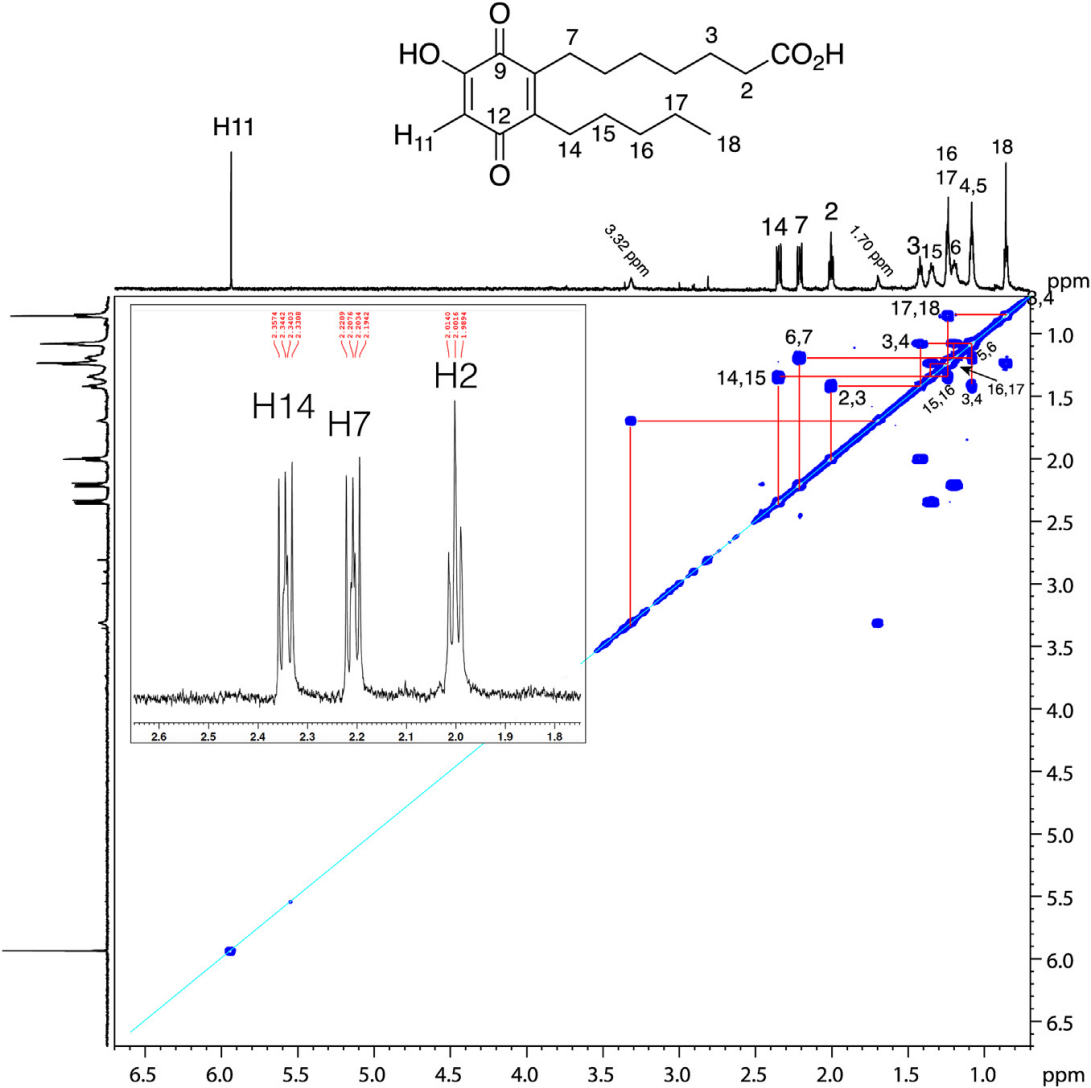
**^1^H-NMR spectrum (600 MHz) and COSY analysis of Peak-2 from**Figure 4**(10-hydroxy-benzoquinone).** The spectrum was recorded in d_6_-benzene. The prominent downfield singlet at 5.93 ppm represents H11; the equivalent proton in a hydroxy-quinone substructure is reported at 5.81 in CDCl_3_ solvent (35). We have not assigned the two coupled signals at 1.7 and 3.2 ppm; their chemical shifts do not match common impurities (36) and their areas are each around half of other protons in the spectrum. Inset: detailed view of H14, H7 and the triplet of H2.

Proton NMR with COSY analysis allowed assignment of Peak-2 as a C18 hydroxy-benzoquinone fatty acid, Fig. 5. The NMR spectrum has a one proton singlet at 5.93 ppm, not coupled to other protons and assigned as H11, the only proton on a carbon of the 6-membered ring. Other diagnostic signals are the two side-chain methylene groups alpha to the ring (H14 at 2.34 ppm, and H7 at 2.21 ppm), which as expected couple to upfield protons on the side chains. H14 and H7 share an unusual multiplicity with three equal-sized peaks and with smaller internal signals around the center, and possibly including long-range coupling to each other, Fig. 4, inset. Based on the UV, LC-MS, NMR and formation from authentic hydroxy-cyclohexenone, Peak-2 is assigned as a 10-hydroxy-benzoquinone C18 fatty acid (Fig. 5). We conclude that alkali degrades 9,10-dihydroxy-11*E*-13-ketone to the pair of hydroxy-cyclohexenones, and under the conditions using purified starting material, most of the second 10-oxo-11-hydroxy isomer is recovered as the oxidized Peak-2, the hydroxy-benzoquinone.

### Effects of mild alkali treatment of the thiolate conjugates of epoxy-ketone and dihydroxy ketone

**Figure 6:**
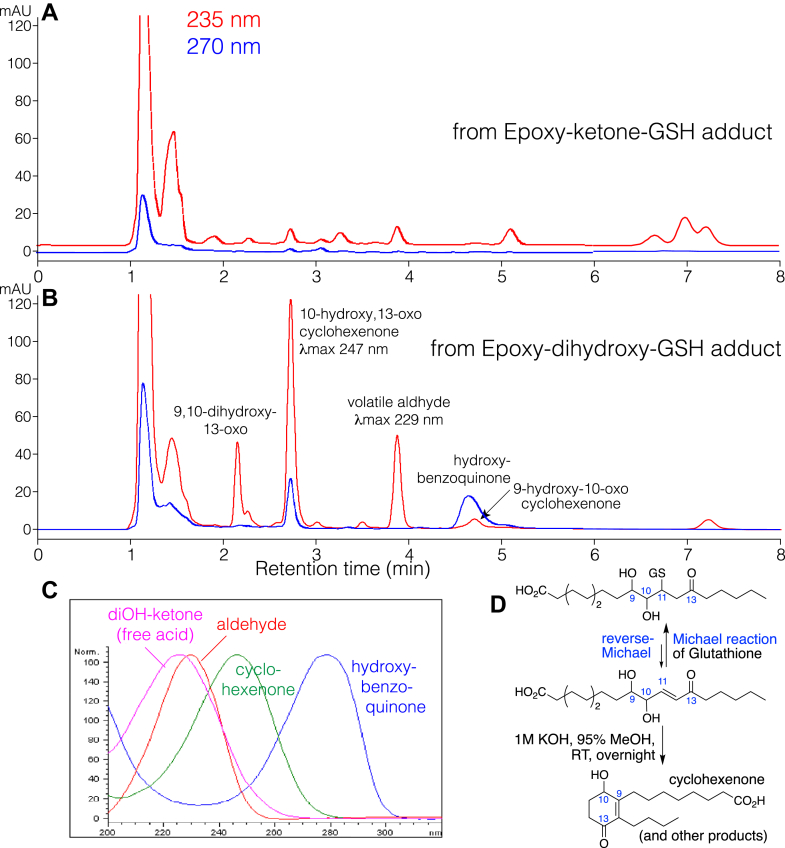
**RP-HPLC analysis of products from KOH treatment of the glutathione adducts of linoleate 9,10-*trans*-epoxy-11*E*-13-ketone and 9,10-dihydroxy-11*E*-13-ketone.** Aliquots (∼5 μg) of the two glutathione conjugates were treated with 20 μl of 95% methanolic KOH overnight at room temperature, then 50 μl of 3% aqueous acetic acid added, and 20% injected on an Agilent Eclipse 5 μ XDB-C18 column (15 × 0.46 cm) with a solvent of acetonitrile/water/glacial acetic acid (60:40:0.01 by volume) at a flow rate of 1 ml/min, with UV diode array detection. *Panel* A: profile of products from KOH-treated 9,10-*trans*-epoxy-11*E*-13-ketone GSH adduct. *Panel* B: profile of products from KOH-treated 9,10-dihydroxy-11*E*-13-ketone GSH adduct. *Panel* C: UV spectra of products from *panel* B. Panel D. Reverse Michael addition of 9,10-dihydroxy-11*E*-13-ketone GSH adduct leads to cyclohexenone and other products.

It is deduced that in the mouse skin barrier, a major mechanism of covalent binding of ceramides is the adduction of Cer-EOS-epoxy-ketone to thiol residues in the protein (20). To test whether thiol-adducted epoxy-ketone could be a source of the two hydroxy-cyclohexenones in vitro, we alkali-treated the synthetic glutathione conjugates and examined the products formed by HPLC-UV. Again, starting with epoxy-ketone-GSH adducts, formation of the two cyclic derivatives was not detected, Fig. 6A, although other UV-absorbing products formed by KOH treatment of the free epoxy-ketone were evident (e.g., the group of three peaks at ∼7 min, cf. Fig. 4, Figure 6A).

Starting with a similar quantity of dihydroxy-ketone-GSH conjugate, KOH treatment produced a familiar pattern of hydroxy-cyclohexenones and hydroxy-benzoquinone, Fig. 6B. Also present on the chromatogram are the free acid of intact dihydroxy-ketone at 2.5 min, and a volatile product with UV λmax 229 nm at 3.9 min; the latter was not identified, although its volatility suggests it could be aldehydic in structure. We conclude that exposure of the dihydroxy-ketone-GSH conjugate to alkali gives the same “polar” lipid recovered from KOH-treated skin barrier proteins, while the conditions appear to favor oxidation of the “less polar” lipid to the hydroxy-benzoquinone.

### Yield of the hydroxy-cyclohexenones

**Fig. 7:**
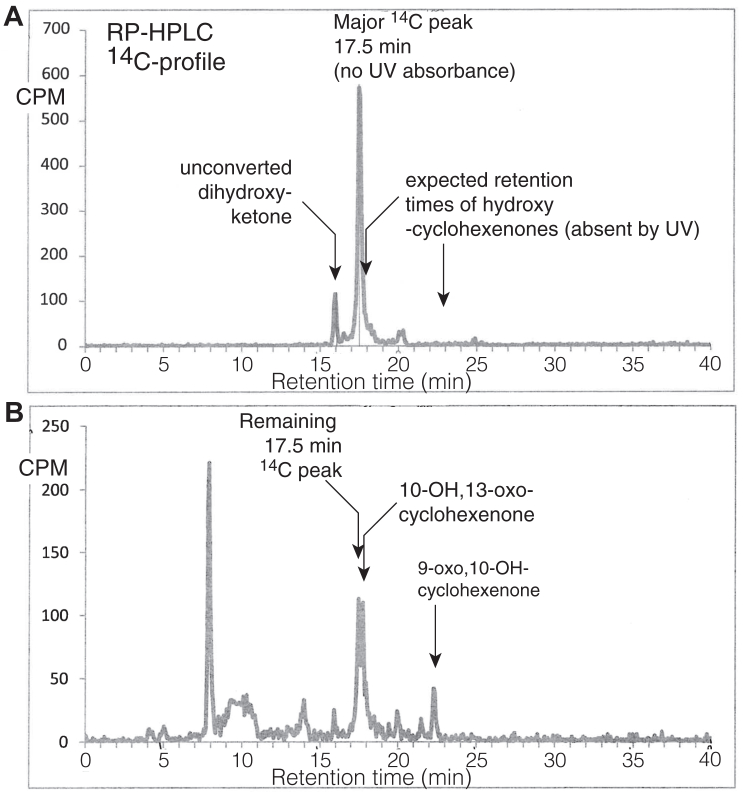
**RP-HPLC analyses of methanolic KOH treatment of [1–^14^C]9,10-dihydroxy-11*E*-13-ketone.** A: Radiochromatogram (with simultaneous UV detection at 205, 220, 235 and 270 nm, not shown) of the products from [1–^14^C]9,10-dihydroxy-11*E*-13-ketone after 10 min in 90% methanolic 1M KOH at room temperature. B: Radiochromatogram after overnight exposure in 90% methanolic KOH (21 h). Samples were run on an Agilent XDB-C18 column (150 × 4.6 mm) with a solvent gradient of acetonitrile/water/glacial acetic acid in the proportions 20:80:0.01 changing to 80:20:0.02 over 30 min and then held to the end of the run. A repeat experiment gave essentially the same results.

**Fig. 8:**
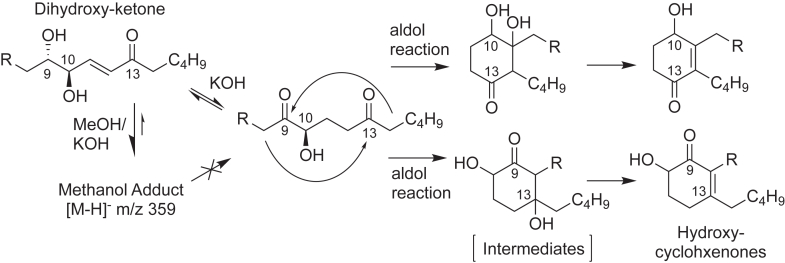
**KOH-induced transformations of linoleate 9,10-dihydroxy-11*E*-13-ketone**. *Left* side: Exposure to methanolic KOH promotes adduction of methanol (likely on the 11-carbon) forming the major peak detectable using ^14^C-labeling (Fig. 7A) and by LC-MS (*m/z* 359, accompanying text). KOH also induces ketone-enol tautomerism to a diketo intermediate (illustrated in ref (19)) then intramolecular aldol reactions leading to two transient hydroxylated intermediates, with spontaneous dehydration to the two hydroxy-cyclohexenones. R = (CH_2_)_6_-CO_2_H.

As evidenced in Figs. 4 and 6, hydroxy-cyclohexenone formation is readily detectable by HPLC-UV analysis after overnight treatment of 9,10-*erythro*-dihydroxy-11*E*-13-ketone with 90% methanolic 1M KOH. Nonetheless, it became apparent that the recovered yield is low, in the order of 5%–10% as estimated from the UV absorbance of the starting material and products. We prepared ^14^C-labeled dihydroxy-ketone and examined the RP-HPLC profile after 10 min treatment with 90% methanolic KOH, a time-point when most of the dihydroxy-ketone is transformed yet before appearance of the two hydroxy-cyclohexenones. As shown in Fig. 7A, a major radiolabeled peak, undetectable by UV absorbance, appears just after elution of the remaining dihydroxy-ketone starting material. This was identified on a Thermo Q Exactive LC-MS as having an [M-H]^-^ ion and base peak at *m/z* 359.2430, which is within 2.5 ppm of the exact mass of the dihydroxy ketone [M-H]^-^ ion (*m/z* 327.2177) plus the exact mass of methanol (32.0262); *m/z* 359 readily gives *m/z* 327 as a product ion (and the two chromatographic profiles match). We conclude that methanolic KOH promotes addition of methanol to the dihydroxy-ketone (Fig. 8, left side); the adduct is in equilibrium with the free dihydroxy-ketone and slows alkali-induced conversion to the two hydroxy-cyclohexenones (Fig. 8, left side). After overnight exposure to KOH, the polar hydroxy-cyclohexenone and the hydroxy-benzoquinone are present on the radiochromatogram (Fig. 7B and Fig. 8, right side) along with additional radiolabeled peaks including a prominent cleavage product eluting at ∼8 min; it gives an [M-H]^-^ ion and base peak at *m/z* 187.0967 (C_9_H_15_O_4_^-^, calc. 187.0976) corresponding to a C9 dicarboxylic acid including retention of the ^14^C-labeled carboxyl.

### Quantitation of hydroxy-cyclohexenones from wild-type and Sdr9c7^−/−^ epidermis

**Fig. 9:**
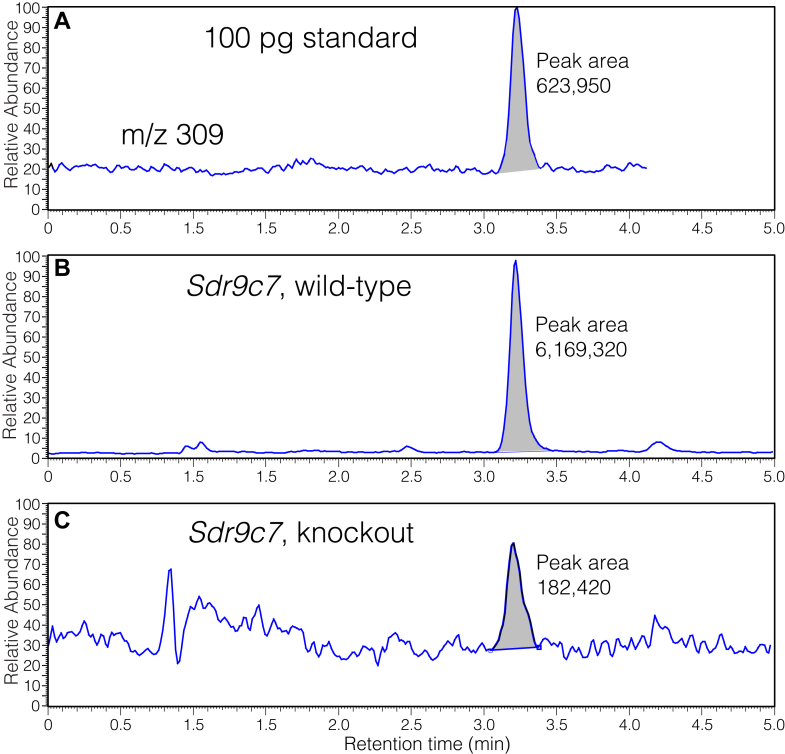
**LC-MS analysis of 10-hydroxy-13-oxo-cyclohexenone recovered after KOH treatment of epidermal proteins from wild-type and knockout *Sdr9c7* mice.** Mouse pup epidermis were weighed, washed extensively with MeOH/CHCl_3_ to remove free lipids, then treated overnight with 95% methanolic 1M KOH followed by neutralization and solvent extraction. Samples were analyzed by negative ion APCI with monitoring of the [M-H]^−^ ion at *m/z* 309 using a Waters 5 μ C18 Symmetry column (150 × 2.1 mm) with a solvent of acetonitrile/water/glacial acetic acid (50:50:0.01 by volume) at a flow rate of 0.3 ml/min. The three panels show (A), injection of 100 pg authentic hydroxy-cyclohexenone (10-hydroxy-13-oxo), and product from (B) *Sdr9c7* mouse epidermis wild-type and (C) *Sdr9c7* deficient epidermis.

The premise that the two hydroxy-cyclohexenones are derived from derivatives of the 12*R*-LOX pathway implies their KOH-induced formation should be reduced or eliminated in epidermis of the knockout mice. Accordingly, we quantified the KOH-induced production of the “polar” hydroxy-cyclohexenone from wild-type and knockout *Sdr9c7* epidermal proteins (Fig. 9). The results showed major reductions in its formation in the *Sdr9c7* knockout (0.11 ± 0.016 (SEM) ng/mg protein in the knockouts (n = 5) compared to 0.99 ng/mg in wild-type and heterozygotes, two-tailed *P* value 0.033). While the levels were reduced to about 10% of normal in comparison to the wild-type and heterozygous tissue, the LC-MS method used for their detection and quantitation demonstrated low levels detectable also in the knockout epidermis, Fig. 9, a point considered in the Discussion.

## Discussion

Multiple lines of evidence indicate that a primary role of the 12*R*-LOX pathway enzymes in the epidermis is to facilitate the covalent binding of acylceramides to the skin barrier protein (10, 12, 20). This forms the CLE, a coat of lipid on the outer surface of the corneocytes, and an integral sub-structure of the permeability barrier (7). The linoleate oxidations promoted by 12*R*-LOX, eLOX3 and SRD9C7 result in formation of a conjugated enone moiety that reacts readily with the thiolate of cysteine residues (Fig. 2) (20). Indeed, as the quantitative analysis of reaction rates establish, the enone moiety of the 9,10-*trans*-epoxy-11*E*-13-ketone reacts 100-fold faster with thiols than the enone of a prototypical unsaturated fatty acid ketone such as 13-oxo-ODE. This in itself might rationalize having three enzymes of the 12*R*-LOX pathway to produce the reactive epoxy-ketone.

The non-enzymic coupling to thiols might be sufficient to account for covalent binding of the oxidized acylceramides to protein, especially as there can be exceptionally high reactivity of specific Cys residues in protein (eg ref (37)). As an alternative, a potential enzymatic coupling could be the means for direction to specific targets. Another consideration is the last step of oxidation in the 12*R*-LOX pathway is the NAD-dependent oxidation of linoleate epoxy-alcohol to epoxy-ketone by SDR9C7, and the NAD-dependence all but necessitates the transformation occurring in a living cell. An enzyme might then direct the product to couple with protein, rather than, for example, with glutathione in the cytosol of living cells. Conceivably, the lipophilicity of the oxidized acylceramide could position the molecule in a hydrophobic environment, thus protected from thiol cofactors in the cytosol.

Lipids of the epidermal barrier covalently bound to protein were characterized in the late 1980s after KOH ester hydrolysis as a mixture of omega-hydroxy-ceramides and hydroxy-fatty acids (21, 22, 23, 38). Thereafter, this mild alkaline hydrolysis is used commonly to quantify components of the covalently bound lipids (e.g., (7, 10, 13, 14, 17, 20, 39, 40)). Recent analysis of the hydroxy fatty acids revealed the formation and release of the two unusual C18 hydroxy-cyclohexenones from the covalently bound lipids, and the evidence points to these being related to the 12*R*-LOX pathway oxidations of acylceramides (19). They might arise from oxidized linoleate in covalently bound acylceramide or acyl acid (41, 42). To further examine this relationship, we measured the abundance of the 10-hydroxy-13-oxo cyclohexenone in KOH-treated epidermal proteins of wild-type and *Sdr9c7* deficient mice. The levels were markedly lower in the knockouts, although remained detectable at approximately 10% of the wild-type and heterozygotes. This confirms that these products are derived largely from enzymatic oxidations of linoleate, although there is a small percentage arising from other routes, perhaps including from non-enzymic linoleate oxidations in the epidermis. For comparison, the epoxy-ketone product produced via Sdr9c7-catalyzed oxidation was 95% reduced but not eliminated in epidermis from the *Sdr9c7* mouse knockout (12).

When the two hydroxy-cyclohexenones formed by KOH from the bound lipids on skin barrier proteins were first characterized, it was speculated they could be derived from a linoleate 9,10-dihydroxy-13-ketone via alkali-induced ketone-enol tautomerism and subsequent intramolecular aldol reactions (19). Here, their mechanistic origin was tested directly and by comparison of the KOH-induced products from authentic 9,10-*trans*-epoxy-ketone, its hydrolysis product 9,10-*erythro*-dihydroxy-13-ketone and their glutathione conjugates. The results establish that the hydroxy-cyclohexenones are not produced from the epoxy-ketone or from its cysteinyl conjugate (GSH adduct). By contrast, they are formed by KOH treatment of the dihydroxy-ketone and its cysteinyl adducts (Figs. 4 and 7), thus giving rise to the same hydroxy-cyclohexenones formed by alkali from epidermal proteins, Fig. 9. The implication is that linoleate 9,10-dihydroxy-11*E*-13-ketone is one of the lipids covalently bound to skin barrier protein.

There is a difference in the HPLC profiles of hydroxy-cyclohexenones recovered from KOH treatment of epidermal protein and from synthetic precursors. While the “polar” 10-hydroxy-13-oxo analogue is represented in both cases, from purified precursor most of the 9-oxo-10-hydroxy analog (earlier referred to as the “less polar” isomer (19)) is recovered as a hydroxy-benzoquinone. This conversion is mimicked by alkali treatment of the purified 9-oxo-10-hydroxy analogue (Supplemental Fig. S6). Benzoquinones are considerably more reactive than unsaturated carbonyls (43), and it is likely the hydroxy-benzoquinone reacts in the proteinaceous environment, resulting in its absence from the HPLC profile of lipid products from KOH-treated barrier proteins.

There is now a strong compendium of evidence linking the 12*R*-LOX oxidations in epidermis with the covalently bound precursors of the two hydroxy-cyclohexenones released after alkali treatment of skin barrier protein. The list includes the hydroxy-cyclohexenones being C18 fatty acids, matching the availability of linoleate in the outer epidermis, they are ^14^C-labeled after incubation of epidermis ex vivo with [^14^C]linoleic acid, and now we establish they are formed by alkali treatment of linoleate dihydroxy-ketone, thus solidly placing the hydroxy-ketone among the covalently bound lipids of the CLE. Quantitative aspects related to their abundance relative to other bound lipids remain to be established.

## Data availability

All data will be made available upon reasonable request.

## Supplemental data

This article contains supplemental data.

## Conflict of interest

The authors declare that they have no conflicts of interest with the contents of this article.
