# Supplementary material for: The origin of hydroxy-cyclohexenone fatty acids from skin barrier protein and relevance to covalent binding of ceramides
Source: J Lipid Res. 2025 Jun 14;66(7):100843. doi: 10.1016/j.jlr.2025.100843 (PMC12274931; doi:10.1016/j.jlr.2025.100843)
Supplement: Supplementary Material [file mmc1.pdf]

Supporting Information

The origin of hydroxy-cyclohexenone fatty acids from skin barrier protein and relevance to covalent binding of ceramides

Saori Noguchi, William E. Boeglin, Fumie Nakashima, Donald F. Stec, M. Wade Calcutt, Takuya Takeichi, Masashi Akiyama and Alan R. Brash

Contents:

Supplemental Figure S1: Analysis of pyrrole adduct of lysine with 9,10-epoxy-11*E*-oxo-octadecenoate substrate
A: Reaction of the epoxy-ketone methyl ester with lysine was conducted in acetonitrile/water (pH 10) and an aliquot analyzed on a Waters Symmetry® C18 5µm column (25 x 0.46 cm) with isocratic elution using MeOH/H2O:80/20 +10 mM Ammonium acetate with a flow rate of 1 ml/min with diode array UV detection at 205, 220, 235 and 270 nm. Inset: UV spectra of the epoxy-ketone and the pyrrole product. B: LC-MS-ESI (negative ion) analysis of the reaction, with the fatty acid pyrrole adduct with $[M\text{-}H]^-$ ion at m/z 451.

Supplemental Figure S2: ^{1}H-NMR (600 MHz) and COSY spectrum recorded in d_4-methanol of the lysine pyrrole adduct of 9,10-epoxy-11*E*-13-oxo-octadecenoic acid

Supplemental Figure S3: Rates of reaction of linoleate epoxy-ketone and dihydroxy-ketone with amino acids at pH 8.5
Reactions were conducted in 0.1 M potassium phosphate pH 8.5, recording at 240 nm to avoid UV saturation effects due to the absorbance of amino acids at lower wavelengths and correcting rates to the respective λmax, 235 nm for 9,10-*trans*-epoxy-11*E*-13-oxo-octadecenoic acid and 228 nm for 9,10-dihydroxy-11*E*-13-oxo-octadecenoic acid (Experimental Procedures). Initial rates after addition of 1 mM cysteine (final concentration) are illustrated. Lysine (1 mM, dotted green trace) and histidine (1mM, dotted black) gave no observable reaction over the time course.

Supplemental Figure S4: LC-MS analysis of adducts of 9,10-*trans*-epoxy-11*E*-13-oxo-octadecenoic acid with L-cysteine
The adducts were chromatographed on a Kinetex 5 µm C18 column (100 x 3 mm) with a solvent of MeOH/H_2O-10 mM NH_4Ac, pH 5.0 at a flow rate of 0.3 ml/min. Mass spectra were recorded on a Thermo Q Exactive HF hybrid quadrupole/orbitrap scanning in negative ESI over a mass range of m/z 100 – 700. The two epoxy-ketone-Cys diastereomers elute at 4.5 and 4.8 min with similar mass spectra (illustrated for the first-eluting diastereomer).

Supplemental Figure S5: ^{1}H-NMR spectrum (600 MHz) and COSY analyses of the second conjugate of N-acetyl-cysteine methyl ester with 9*R*,10*R*-*trans*-epoxy-11*E*-13-ketone
Partial proton spectrum (0.7 – 3.6 ppm) and COSY of the second-eluting (RP-HPLC) diastereomeric conjugate of 9*R*,10*R*-epoxy-11*E*-13-oxo-octadecenoate methyl ester with the methyl ester of N-acetyl-cysteine recorded in d_6-benzene. Thiol adduction at C11 of the epoxy-ketone is clearly evidenced. Not included in this partial spectrum are two protons further downfield, the NAC main chain proton, a double triplet at 5.12 ppm, and the NAC NH proton, a doublet at 6.75 ppm. Note that H12 next to the ketone is a two-proton doublet (cf. in the first-eluting conjugate H12 is two separate dd, Figure 3 main text).

Supplemental Figure 6: RP-HPLC analysis of conversion of the "less polar" 9-oxo-10-hydroxy-cyclohexenone to hydroxy-benzoquinone
Purified 9-oxo-10-hydroxy-cyclohexenone was incubated overnight at room temperature in 1M KOH in 95% MeOH, subsequently extracted with DCM at pH 4 and an aliquot analyzed by RP-HPLC using an Agilent Eclipse XDB-C18 5 µm column (15 x 0.46 cm) with an isocratic solvent of CH_3CN/H_2O/HAc (60:40:0.01 by volume) at a flow rate of 1 ml/min with UV detection at 205 nm (pink trace), 220 nm (green), 235 nm (red), and 270 nm (black). The hydroxy-benzoquinone product elutes at 4.5 min, immediately before the 9-oxo-10-hydroxy-cyclohexenone.

A: RP-HPLC-UV

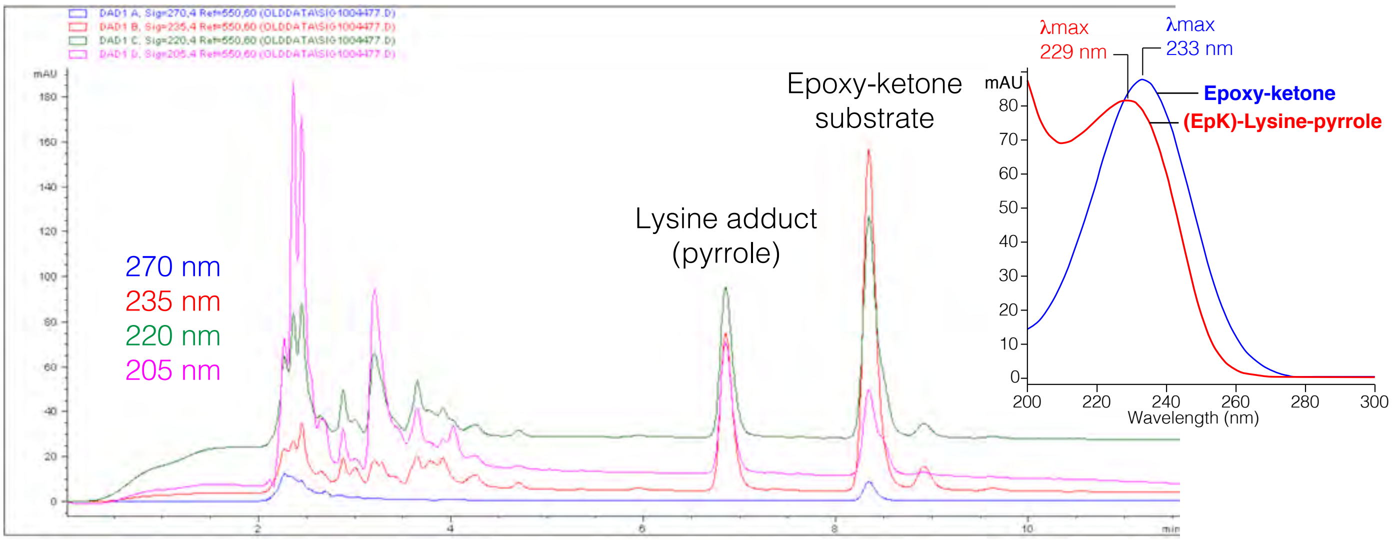

B: LC-MS

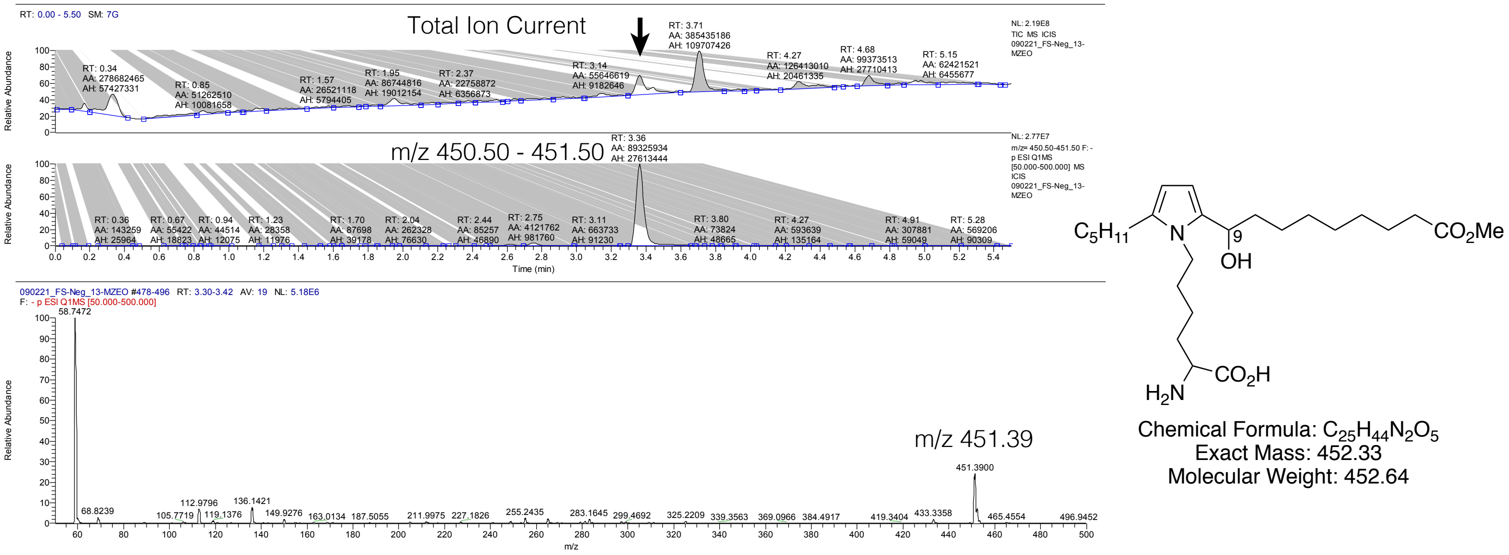

Supplemental Figure S1: Analysis of pyrrole adduct of lysine with 9,10-epoxy-11E-oxo-octadecenoate substrate

A: Reaction of the epoxy-ketone methyl ester with lysine was conducted in acetonitrile/water (pH 10) and an aliquot analyzed on a Waters Symmetry® C18 5μm column (25 x 0.46 cm) with isocratic elution using MeOH/H2O:80/20 +10 mM Ammonium acetate with a flow rate of 1 ml/min with diode array UV detection at 205, 220, 235 and 270 nm. Inset: UV spectra of the epoxy-ketone and the pyrrole product. B: LC-MS-ESI (negative ion) analysis of the reaction, with the fatty acid pyrrole adduct with [M-H]- ion at m/z 451.

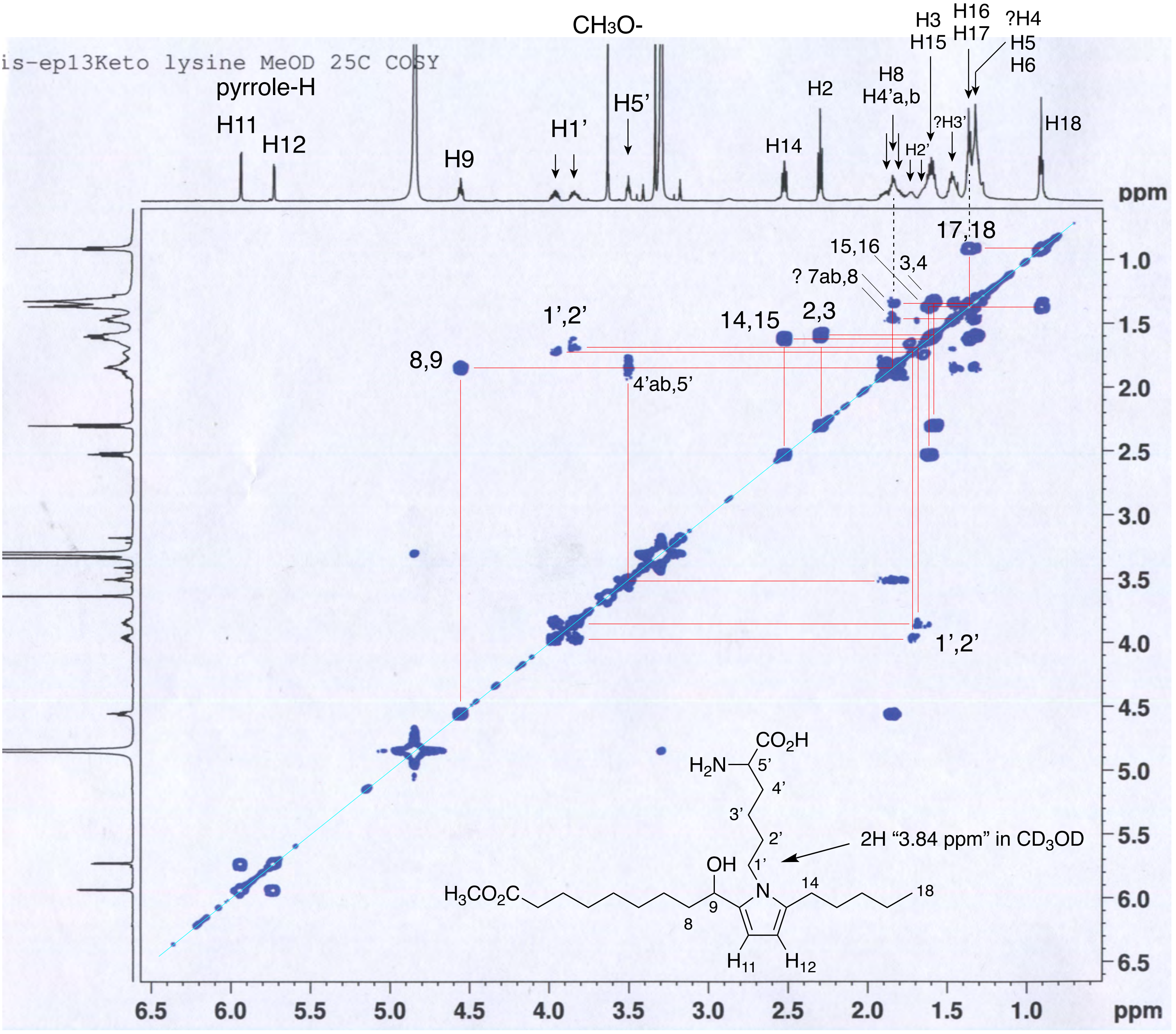

Supplemental Figure S2: ^{1}H-NMR and COSY spectrum recorded in d_4-methanol of the lysine pyrrole adduct of 9,10-epoxy-11*E*-13-oxo-octadecenoic acid.
Inset shows the structure and numbering of protons.

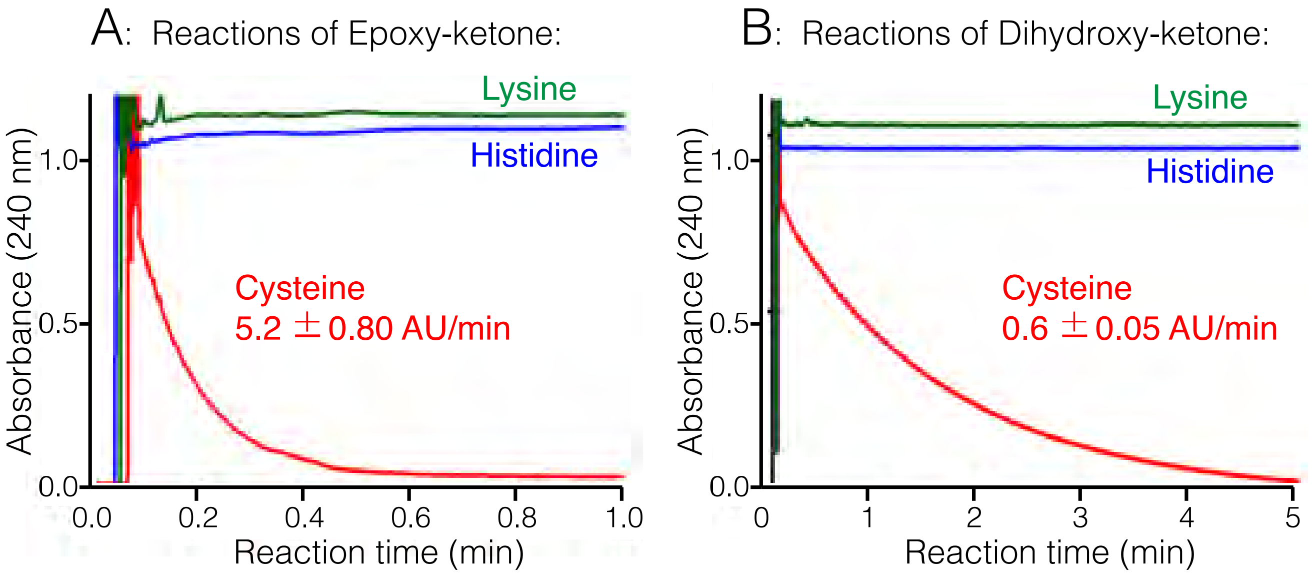

Supplemental Figure S3: Rates of reaction of linoleate epoxy-ketone and dihydroxy-ketone with amino acids at pH 8.5

Reactions were conducted in 0.1 M potassium phosphate pH 8.5, recording at 240 nm to avoid UV saturation effects due to the absorbance of amino acids at lower wavelengths and correcting rates to the respective lambda-max, 235 nm for 9,10-trans-epoxy-11E-13-oxo-octadecenoic acid and 228 nm for 9,10-dihydroxy-11E-13-oxo-octadecenoic acid (Experimental Procedures). Initial rates after addition of 1 mM cysteine (final concentration) are illustrated. Lysine (1 mM, green trace) and histidine (1mM, blue) gave no observable reaction over the time course.

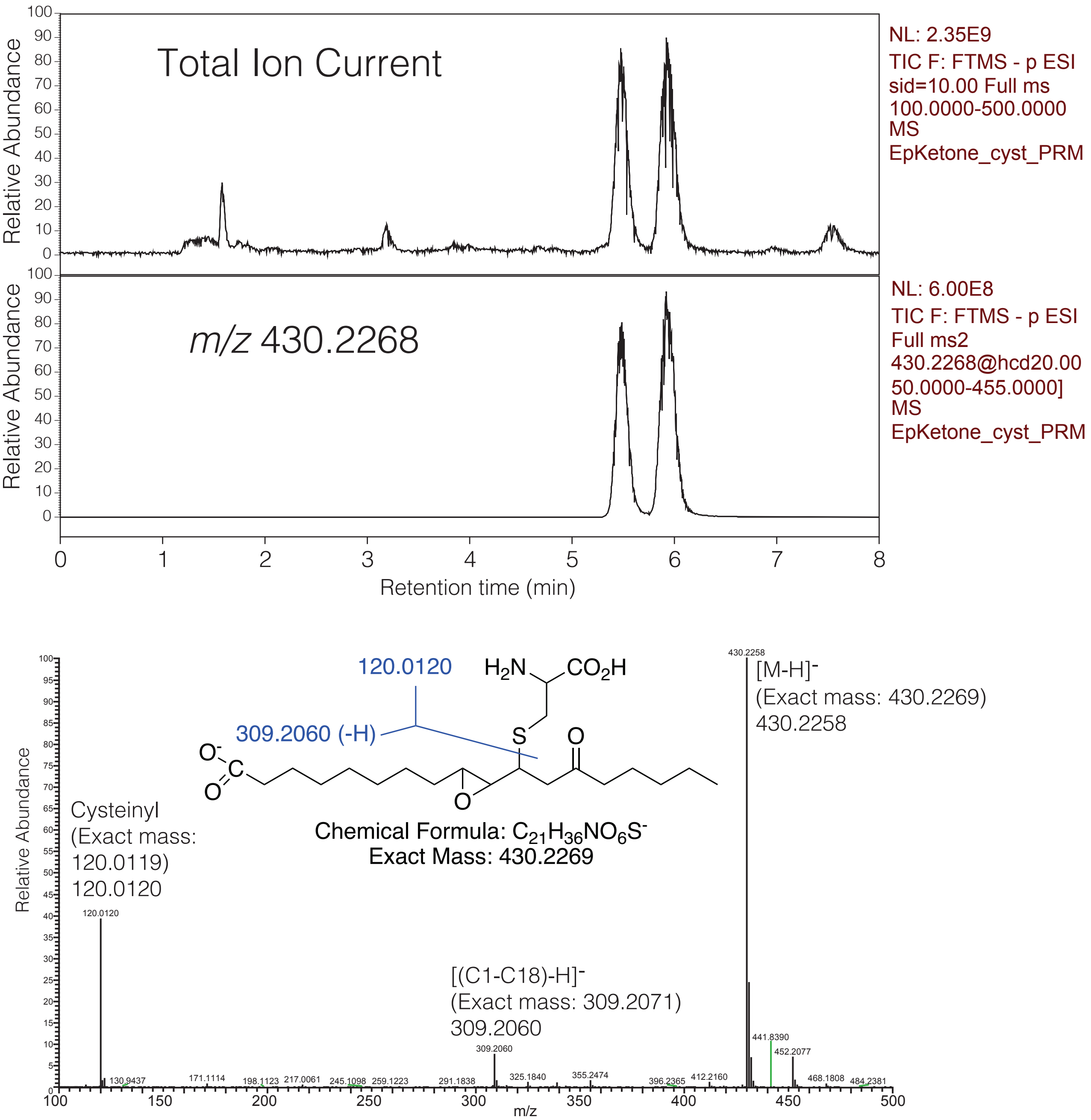

Supplemental Figure S4: LC-MS analysis of adducts of 9,10-*trans*-epoxy-11*E*-13-oxo-octadecenoic acid with L-Cysteine

The adducts were chromatographed on a Kinetex 5 μm C18 column (100 x 3 mm) with a solvent of MeOH/H2O-10 mM NH4Ac, (60:40) pH 5.0 at a flow rate of 0.3 ml/min. Mass spectra were recorded on a Thermo Q Exactive HF hybrid quadrapole/orbitrap scanning in negative ESI over a mass range of m/z 100 – 500. The two epoxy-ketone-Cys diastereomers elute at 5.50 and 5.95 min with similar mass spectra (illustrated for the first-eluting diastereomer).

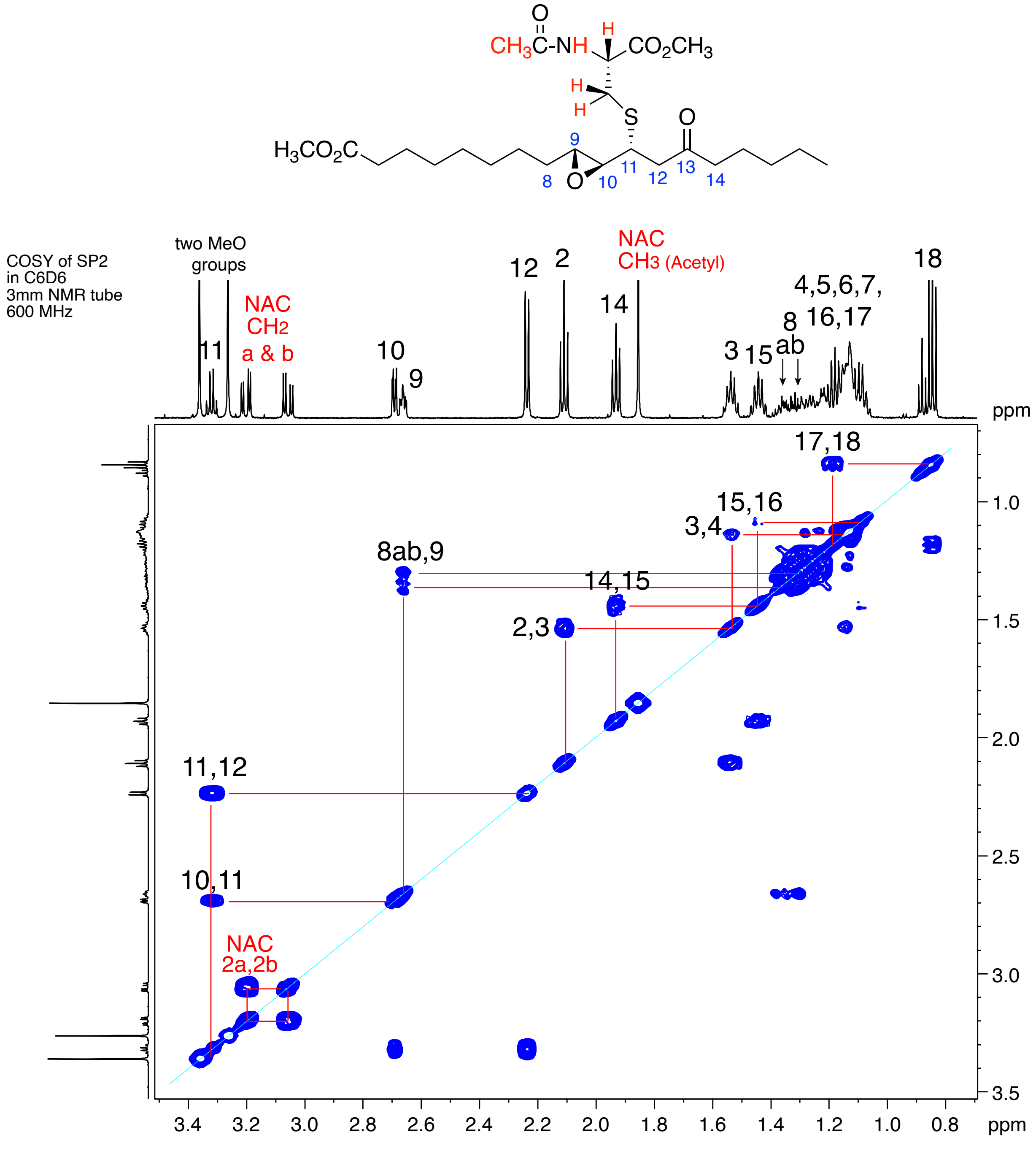

Supplemental Figure S5: ^{1}H-NMR spectrum (600 MHz) and COSY analyses of the second conjugate of N-acetyl-cysteine methyl ester with 9,10-*trans*-epoxy-11*E*-13-ketone
Partial proton spectrum (0.7 – 3.6 ppm) and COSY of the second-eluting (RP-HPLC) diastereomeric conjugate of 9*R*,10*R*-epoxy-11E-13-oxo-octadecenoate methyl ester with the methyl ester of N-acetyl-cysteine recorded in d6-benzene. Not included in this partial spectrum are two protons further downfield, the NAC main chain proton, a double triplet at 5.12 ppm, and the NAC NH proton, a doublet at 6.75 ppm. Note that H12 next to the ketone is a two-proton doublet (cf. in the first-eluting conjugate H12 is two separate dd, Figure 3 main text).

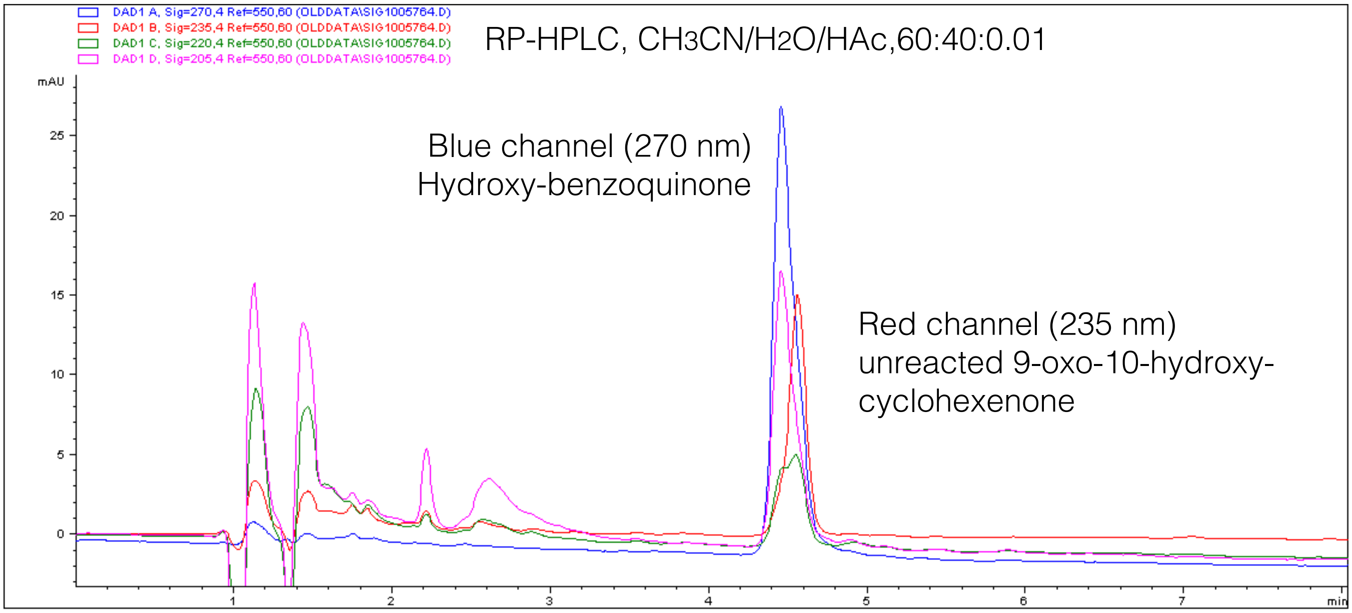

Supplemental Figure 6: RP-HPLC analysis of conversion of the "less polar" 9-oxo-10-hydroxy-cyclohexenone to 10-hydroxy-benzoquinone

Purified 9-oxo-10-hydroxy-cyclohexenone was incubated overnight at room temperature in 1M KOH in 95% MeOH, subsequently extracted with DCM at pH 4 and an aliquot analyzed by RP-HPLC using an Agilent Eclipse XDB-C18 5 um column (15 x 0.46 cm) with an isocratic solvent of $CH_3CN/H_2O/HAc$ (60:40:0.01 by volume) at a flow rate of 1 ml/min with UV detection at 205 nm (pink trace), 220 nm (green), 235 nm (red), and 270 nm (black). The hydroxy-benzoquinone product elutes at 4.5 min, immediately before the hydroxy-cyclohexenone substrate.
